# Improved Quantitative Plant Proteomics via the Combination of Targeted and Untargeted Data Acquisition

**DOI:** 10.3389/fpls.2017.01669

**Published:** 2017-09-27

**Authors:** Gene Hart-Smith, Rodrigo S. Reis, Peter M. Waterhouse, Marc R. Wilkins

**Affiliations:** ^1^NSW Systems Biology Initiative, School of Biotechnology and Biomolecular Sciences, University of New South Wales, Sydney, NSW, Australia; ^2^School of Biological Sciences, University of Sydney, Sydney, NSW, Australia; ^3^Department of Plant Molecular Biology, University of Lausanne, Lausanne, Switzerland; ^4^Centre for Tropical Crops and Biocommodities, Queensland University of Technology, Brisbane, QLD, Australia

**Keywords:** quantitative plant proteomics, targeted data acquisition (TDA), data-dependent acquisition (DDA), metabolic ^15^N-labeling, liquid chromatography-tandem mass spectrometry (LC-MS/MS), *Arabidopsis thaliana*

## Abstract

Quantitative proteomics strategies – which are playing important roles in the expanding field of plant molecular systems biology – are traditionally designated as either hypothesis driven or non-hypothesis driven. Many of these strategies aim to select individual peptide ions for tandem mass spectrometry (MS/MS), and to do this mixed hypothesis driven and non-hypothesis driven approaches are theoretically simple to implement. In-depth investigations into the efficacies of such approaches have, however, yet to be described. In this study, using combined samples of unlabeled and metabolically ^15^N-labeled *Arabidopsis thaliana* proteins, we investigate the mixed use of targeted data acquisition (TDA) and data dependent acquisition (DDA) – referred to as TDA/DDA – to facilitate both hypothesis driven and non-hypothesis driven quantitative data collection in individual LC-MS/MS experiments. To investigate TDA/DDA for hypothesis driven data collection, 7 miRNA target proteins of differing size and abundance were targeted using inclusion lists comprised of 1558 *m/z* values, using 3 different TDA/DDA experimental designs. In samples in which targeted peptide ions were of particularly low abundance (i.e., predominantly only marginally above mass analyser detection limits), TDA/DDA produced statistically significant increases in the number of targeted peptides identified (230 ± 8 versus 80 ± 3 for DDA; *p* = 1.1 × 10^-3^) and quantified (35 ± 3 versus 21 ± 2 for DDA; *p* = 0.038) per experiment relative to the use of DDA only. These expected improvements in hypothesis driven data collection were observed alongside unexpected improvements in non-hypothesis driven data collection. Untargeted peptide ions with *m/z* values matching those in inclusion lists were repeatedly identified and quantified across technical replicate TDA/DDA experiments, resulting in significant increases in the percentages of proteins repeatedly quantified in TDA/DDA experiments only relative to DDA experiments only (33.0 ± 2.6% versus 8.0 ± 2.7%, respectively; *p* = 0.011). These results were observed together with uncompromised broad-scale MS/MS data collection in TDA/DDA experiments relative to DDA experiments. Using our observations we provide guidelines for TDA/DDA method design for quantitative plant proteomics studies, and suggest that TDA/DDA is a broadly underutilized proteomics data acquisition strategy.

## Introduction

Quantitative proteomics studies are playing a crucial role in the advancing field of plant molecular systems biology. These studies make use of quantitative data for peptides, collected using liquid chromatography (LC)-tandem mass spectrometry (MS/MS), to measure relative protein abundances across samples. In this manner they can facilitate the study of protein expression levels across, for example, different stages of plant development, tissue types, genotypes, physiological conditions and stress conditions (Arsova et al., 2012a; Hu et al., 2015; Jorrín-Novo et al., 2015), and thus offer a potent means for gaining insight into the molecular underpinnings of plant biology.

Quantitative proteomics studies are often categorized as either hypothesis driven or non-hypothesis driven (Domon and Aebersold, 2010). Hypothesis driven studies analyze specific sets of targeted proteins known or hypothesized to be of biological interest (Schmidt et al., 2009; Gillet et al., 2016). Studies of this nature have, for example, been used to assess the stress tolerance of field-grown crops (Jacoby et al., 2013), and gain insight into the molecular underpinnings of reactive oxygen species signaling in *Arabidopsis thaliana* (Arabidopsis) (Konert et al., 2015). To ensure that each targeted protein is identified and quantified, hypothesis driven studies acquire MS/MS data for peptides associated with these proteins in either a targeted manner (e.g., using selected reaction monitoring (SRM) (Picotti and Aebersold, 2012), parallel reaction monitoring (PRM) (Peterson et al., 2012) or targeted data acquisition (TDA) (Schmidt et al., 2008; Domon et al., 2009; Savitski et al., 2010; Hart-Smith et al., 2012), or via comprehensive data independent acquisition (DIA) strategies [e.g., sequential window acquisition of all theoretical spectra (SWATH) (Gillet et al., 2012)], which produce quantitative data that must be extracted using protein assay libraries (Schubert et al., 2015). Hypothesis driven studies are capable of quantifying specific proteins with unparalleled sensitivity and selectivity (Picotti et al., 2009). However, they are either incapable of collecting broad-scale quantitative data (i.e., when data is collected in a targeted manner), or in the case of DIA-derived data, remain limited in their accuracy when broad-scale quantification is attempted using large assay libraries (Wu et al., 2016).

Non-hypothesis driven studies, in contrast, are exploratory in nature. They aim to identify and quantify as many proteins as possible in an untargeted manner. This is generally achieved using data dependent acquisition (DDA) during LC-MS/MS, in which the highest abundance peptide ions from full MS scans are selected for MS/MS (Kalli et al., 2013). Non-hypothesis driven studies have generated in-depth molecular insights into numerous aspects of plant biology, from early leaf senescence (Hebeler et al., 2008) and osmotic stress tolerance (Skirycz et al., 2011) to microRNA (miRNA) regulated gene expression (Reis et al., 2015b). However, these studies can be limited by the fact that DDA produces datasets skewed toward the identification of relatively high abundance proteins (Michalski et al., 2011); low abundance proteins of particular biological interest may therefore be excluded from quantification.

**FIGURE 1:**
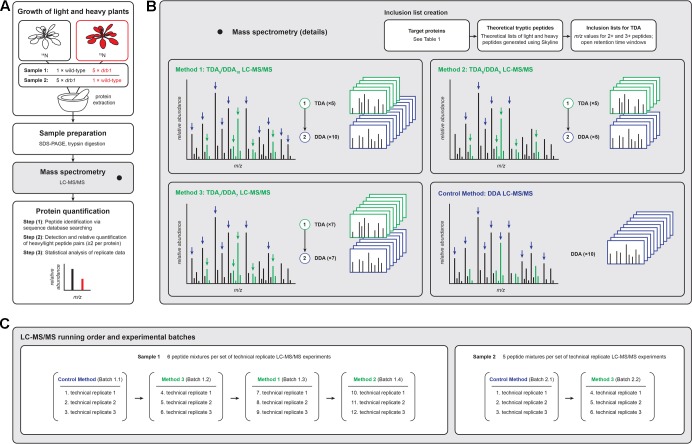
Experimental workflows for the LC-MS/MS-based relative quantification of plant proteins used in the present investigation. **(A)** Proteins from unlabeled ‘light’ and ^15^N-labeled ‘heavy’ plants were mixed, jointly prepared for LC-MS/MS analysis, and the data analyzed following established procedures [see Ting et al. (2009) and Reis et al. (2015a) for detailed descriptions of the statistical procedures required to quantify proteins using these data]. **(B)** TDA/DDA LC-MS/MS methods employed TDA inclusion lists for the hypothesis driven selection of peptides for MS/MS, followed by DDA for non-hypothesis driven data collection; the LC-MS/MS control method employed DDA only. Green MS signals represent peptides derived from targeted proteins, and black MS signals represent other peptides. Heavy peptides (colored red in **A**) are not differentially colored in **B**. **(C)** Batches of LC-MS/MS experiments were performed, with each batch comprised of three sets of technical replicate TDA/DDA or DDA experiments. Proteolytic peptide mixtures derived from sample 1 were analyzed using all TDA/DDA and DDA methods; proteolytic peptide mixtures derived from sample 2 were analyzed using TDA_7_/DDA_7_ (method 3) and the DDA control method only.

Despite this traditional segregation of quantitative proteomics into hypothesis and non-hypothesis driven studies, mixed approaches are possible. It can for example be envisaged that mixed targeted and untargeted MS/MS data collection strategies – which seek to keep the advantages of both hypothesis driven and non-hypothesis studies – should be of widespread utility (including in LC-MS/MS experiments designed to create protein assay libraries for DIA data). For example in recent investigations into miRNA regulated gene expression in Arabidopsis, we used such a strategy to quantify proteins in plants mutant for the proteins DOUBLE-STRANDED RNA-BINDING1 (DRB1) or DRB2, relative to wild-type plants (Reis et al., 2015a,b). A targeted MS/MS strategy, TDA, which employs lists of *m/z* values to be selected for MS/MS even if higher abundance ions are present (inclusion lists), was used to collect data for targeted proteins of hypothesized biological interest (miRNA target proteins). When TDA events were not triggered, MS/MS data was collected from the same experiment using DDA. (This mixed use of TDA and DDA, illustrated in **Figure 1** and elaborated upon in the section “Materials and Methods,” is henceforth referred to as TDA/DDA.) The hypothesis driven data helped show that DRB2 determines miRNA-guided translation inhibition (Reis et al., 2015a), while the non-hypothesis driven data allowed proteome-scale changes in protein expression to be concomitantly studied, revealing unanticipated roles for and secondary effects of translation inhibition in Arabidopsis (Reis et al., 2015b).

Although mixed hypothesis and non-hypothesis driven approaches to MS/MS data acquisition are simple to implement, in-depth investigations into the efficacies of such approaches have yet to be described. In particular it is not known if such mixed approaches compromise non-hypothesis driven data collection relative to standard DDA methods. In the present contribution, in an extension of our previous studies (Reis et al., 2015a,b), we perform these investigations for TDA/DDA in a model quantitative plant proteomics experiment employing the metabolic ^15^N-labeling strategy (Arsova et al., 2012a), using combined samples of unlabeled and ^15^N-labeled wild-type and *drb1* Arabidopsis (**Figure 1**). Specifically we investigate the efficacies of hypothesis and non-hypothesis driven LC-MS/MS data collection using different TDA/DDA experimental designs, relative to data collection using DDA only. Previous studies into the design and efficacy of TDA (e.g., Schmidt et al., 2008; Domon et al., 2009; Savitski et al., 2010; Hart-Smith et al., 2012) and DDA (e.g., Andrews et al., 2011; Michalski et al., 2011; Kalli and Hess, 2012; Kalli et al., 2013) have focused on their use as stand-alone data acquisition techniques. This study therefore provides the first in-depth investigation into the performances of these data acquisition strategies when they are used in combination. Using our observations we provide guidelines for the design of TDA/DDA LC-MS/MS methods for use in quantitative plant proteomics experiments.

## Materials and Methods

**Figure 1** provides an overview of the present study’s experimental design. **Figure 1A** summarizes the overall sample preparation and analysis workflow, **Figure 1B** the TDA/DDA and DDA LC-MS/MS methods studied, and **Figure 1C** the LC-MS/MS experimental batches and running order. Details pertaining to each of these aspects of the study are presented below.

### Plant Lines and Growth Conditions

The wild-type and *drb1* Arabidopsis plants studied here show differential accumulation of proteins influenced by miRNA-guided transcript cleavage (Reis et al., 2015a,b), and thus provide a model system for studying quantitative changes in the proteome. The *drb1* T-DNA knockout insertions have been described previously (Curtin et al., 2008; Eamens et al., 2012). Plant lines were cultivated under standard growth conditions of 16 h light/8 h dark at a constant temperature of 24°C. Unlabeled plants were grown on a modified Murashige and Skoog medium containing half nitrogen concentration (0.825 g/L NH_4_NO_3_ and 0.95 g/L KNO_3_) supplemented with 0.4512 g/L KCl to compensate for potassium reduction. Metabolically ^15^N-labeled plants were grown in a medium in which the nitrogen source was replaced with ^15^NH_4_^15^NO_3_ and K^15^NO_3_ (Cambridge Isotope Laboratories Inc.; > 98% enriched in ^15^N). The average ^15^N-labeling efficiency of proteins was determined to be 97.6 (± 0.2) atom%, as calculated using monoisotopic (M) and M-1 peak ratios for a series of high confidence peptide sequence matches (obtained from the LC-MS/MS experiments described below) following Schaff et al. (2008).

### Sample Preparation

As illustrated in **Figure 1A** and described previously (Reis et al., 2015a), shoot apex samples from 4-week-old unlabeled wild-type and ^15^N-labeled *drb1* mutant plants were harvested and mixed (sample 1), and reciprocal samples were produced in the same manner using ^15^N-labeled wild-type and unlabeled *drb1* mutant plants (sample 2). Following guidelines outlined by Arsova et al. (2012b), harvested wild-type and *drb1* shoot apices were mixed at approximately a 1:5 (*w/w*) ratio. This use of uneven reciprocally labeled samples allows specific effects relating to the identification and quantification of ^15^N-labeled peptides to be studied. Extracted proteins from each sample were separated by 1D SDS–PAGE, stained with colloidal Coomassie G-250 and gel lanes were cut into 29 pieces from low to high protein mass. Each polyacrylamide gel slice was destained, reduced and alkylated following the procedure described by Shevchenko et al. (1996). Protein digestion was performed by incubating each gel slice with 40 ng of trypsin (Stratagene, #204310) in 120 μL of 0.1 M NH_4_HCO_3_ at 37°C for 16 h. The resulting solutions were transferred to new microfuge tubes and gel slices treated with the following solutions sequentially for 30 min per treatment: (i) 80 μL 0.1% (*v/v*) formic acid / 67% (*v/v*) acetonitrile, and (ii) 80 μL 100% acetonitrile. Pooled peptide solutions were then dried (Savant SPD1010, Thermofisher Scientific) before resuspension in 20 μL 0.1% (*v/v*) formic acid. Our previous analyses of these samples (Reis et al., 2015a,b) identified the particular gel slices containing the miRNA target proteins of **Table 1** to be subjected to TDA (elaborated upon below). Only proteolytic peptide samples derived from these particular gel slices were subjected to LC-MS/MS.

**Table 1:** miRNA target proteins targeted for relative quantification and details of their associated TDA inclusion lists.

Protein name	Gene symbol	TAIR accession	Molecular weight (Da)	Peptides targeted	Inclusion list size	Application in sample 1	Application in sample 2
ARGONAUTE 1	AGO1	1009110133	116,190	114	453	Peptide mixture #1	Peptide mixture #1
ALDO-KETO REDUCTASE FAMILY 4 MEMBER C8	AKR4C8	1009115294	34,685	39	150	Peptide mixture #6	Peptide mixture #5
ATP SULFURYLASE 1	APS1	1009119867	51,459	46	181	Peptide mixture #5	Peptide mixture #4
ATP SULFURYLASE 3	APS3	1009125796	52,029	29	116	Peptide mixture #5	Peptide mixture #4
GLYCOSYL HYDROLASE FAMILY PROTEIN	BXL7	1009108493	83,891	86	335	Peptide mixture #3	Peptide mixture #3
MYROSINASE-BINDING PROTEIN 1	MBP1	1009105202	50,167	31	118	Peptide mixture #4	Peptide mixture #4
MYROSINASE-BINDING PROTEIN 2	MBP2	1009105204	68,849	52	205	Peptide mixture #2	Peptide mixture #2

### Inclusion List Creation

For hypothesis driven data collection using TDA, seven specific Arabidopsis miRNA target proteins, which cover a range of sizes (35–116 kDa) and abundances, were targeted: AGO1, AKR4C8, APS1, APS3, BXL7, MBP1, and MBP2 (see **Figure 1B** and **Table 1**). In *drb1* Arabidopsis, AKR4C8, MBP1, and MBP2 are expected to show higher accumulation, AGO1 lower accumulation, and APS1, APS3, and BXL7 no significant changes in accumulation relative to wild-type plants (Reis et al., 2015a). Inclusion lists contained *m/z* values associated with theoretical peptide ions of these targeted proteins, created with the aid of Skyline (version 3.1.0.7382, University of Washington). Specifically amino acid sequences for each of the targeted proteins were imported into Skyline, and *m/z* values (<2000 and >350) for doubly and triply charged theoretical peptide ions (unlabeled light and fully ^15^N-labeled heavy) associated with these proteins were generated using the following parameters: Enzyme: Trypsin (1 missed cleavage allowed); Minimum peptide length: 7 amino acids; Maximum peptide length: 25 amino acids; Structural modifications: Carbamidomethyl cysteine; Isotope modifications: ^15^N for all amino acids (when considering heavy peptides only). Exported *m/z* values were inputted into the TDA/DDA methods described below. Only *m/z* values associated with the targeted proteins present in each individual peptide mixture were inputted, as summarized in **Table 1**.

### Mass Spectrometry

**Figure 1B** summarizes the data acquisition methods used in the present LC-MS/MS experiments. For all LC-MS/MS experiments, proteolytic peptide samples were separated by nano-LC using an UltiMate 3000 HPLC and autosampler system (Dionex, Amsterdam, Netherlands), and ionized using positive ion mode electrospray following experimental procedures described previously (Hart-Smith and Raftery, 2012). Briefly, this involved elution of peptides using a linear gradient of H_2_O:CH_3_CN (98:2, 0.1% formic acid) to H_2_O:CH_3_CN (55:45, 0.1% formic acid) at 250 nL/min over 30 min. MS and MS/MS were performed using an LTQ Orbitrap Velos Pro (Thermo Electron, Bremen, Germany) hybrid linear ion trap and Orbitrap mass spectrometer. Survey scans *m*/*z* 350–2000 were acquired in the Orbitrap (resolution = 30,000 at *m*/*z* 400, with an AGC target value of 1,000,000 charges in the linear ion trap (maximum ion injection time = 250 ms); 1 microscan was collected per scan; lock mass was applied to polycyclodimethylsiloxane background ions of exact *m/z* 391.2843 and 445.1200). Peptide ions ( > 5000 counts) with charge states of ≥ 2 were sequentially isolated and fragmented via collision induced dissociation (CID) with an activation *q* = 0.25, an activation time of 30 ms, normalized collision energy of 30% and at an AGC target value of 10,000 charges (maximum ion injection time = 100 ms); 1 microscan was collected per scan and monoisotopic precursor ion selection was enabled. Fragment ions were mass analyzed in the linear ion trap. Dynamic exclusion was applied to ions subjected to MS/MS using the following parameters: repeat count = 1, repeat duration = 30 s and exclusion duration = 45 s.

For TDA/DDA experiments, MS/MS scan cycles were performed as follows: up to the *n* most abundant ions from the inputted inclusion lists were firstly selected for MS/MS using TDA, followed by up to the *m* most abundant ions using DDA. Three different TDA/DDA methods employing the following combinations of TDA and DDA were studied: *n* = 5 and *m* = 10 (method 1: TDA_5_/DDA_10_); *n* = 5 and *m* = 5 (method 2: TDA_5_/DDA_5_); and *n* = 7 and *m* = 7 (method 3: TDA_7_/DDA_7_). Inclusion lists utilized open retention time windows (i.e., retention times covering the entire duration of the LC experiment), and TDA-triggered MS/MS events only occurred in scan cycles featuring eluting peptides with *m/z* values matching those within the employed inclusion lists (± 10 ppm).

For DDA (control method) experiments, MS/MS scan cycles were performed as follows: up to the 10 most abundant ions were selected for MS/MS using DDA.

**Figure 1C** summarizes the different batches of LC-MS/MS experiments performed. Each batch consisted of a given combination of sample and LC-MS/MS method. Sample 1 – comprised of six proteolytic peptide mixtures – was analyzed using each TDA/DDA method and the DDA control method. Sample 2 – comprised of five proteolytic peptide mixtures – was analyzed using TDA_7_/DDA_7_ (method 3) and the DDA control method only (elaborated upon in the section “Results”). Three sets of technical replicate TDA/DDA or DDA LC-MS/MS experiments were performed for each batch, resulting in 102 LC-MS/MS experiments in total.

### Sequence Database Searching and Relative Protein Quantification via Proteome Discoverer

Peak lists derived from LC-MS/MS were submitted to the database search program Mascot (version 2.3, Matrix Science) via Proteome Discoverer (version 1.3, Thermo Scientific). Separate searches were conducted for unlabeled and fully ^15^N-labeled peptides. For unlabeled peptides, the following search parameters were employed: instrument type was default; precursor ion and peptide fragment mass tolerances were ± 5 ppm and ± 0.4 Da, respectively; variable modifications included were carbamidomethyl (C) and oxidation (M); enzyme specificity was trypsin with up to two missed cleavages; and Arabidopsis sequences in the Swiss-Prot database (October 2015 release, 549,646 sequence entries) were searched. For ^15^N-labeled peptides, search parameters were identical to those used for unlabeled peptides, with the following fixed modifications included: ^15^N(1) (A,C,D,E,F,G,I,L,M,P,S,T,V,Y), ^15^N(2) (K,N,Q,W), ^15^N(3) (H) and ^15^N(4) (R). Only peptides deemed to be statistically significant (*p* < 0.05) according to the Mascot expect metric were used for peptide identification and quantification. For the present sequence database searches this corresponds to an average peptide false discovery rate of ∼3% based on Proteome Discoverer *q*-value estimates.

Relative peptide and protein quantification was performed using Proteome Discoverer. Separate search outputs obtained from unlabeled and fully ^15^N-labeled peptide sequence database searches were combined within Proteome Discover to produce consensus quantitative datasets. Relative quantification data was obtained for all peptides observed by Proteome Discoverer to form part of a co-eluting ^15^N-labeled and unlabeled (heavy/light) peptide pair; only one peptide in each heavy/light peptide pair was required to be identified using MS/MS data.

### Feature Detection and Protein Signal Intensity Measurement via MaxQuant

To account for potential differences in peptide ionization efficiencies across batches of LC-MS/MS experiments (i.e., across batches 1.1–1.4 and 2.1–2.2 of **Figure 1C**), two measures of peptide ionization efficiency were obtained for each set of technical replicate LC-MS/MS experiments: total numbers of peptide features, and individual protein signal intensities (i.e., summed peptide ion intensities for individual proteins). These were measured using MaxQuant (version 1.5.8.0), run using standard parameters (Cox and Mann, 2008), and average values determined for each batch of LC-MS/MS experiments.

Peptide features were extracted from the *allpeptides.txt* output.

Protein signal intensities were derived from sequence database searches for unlabeled proteins, performed using Andromeda, with the “match between runs” feature selected. Andromeda searches were performed using the following parameters: precursor ion and peptide fragment mass tolerances were ± 4.5 ppm and ± 0.5 Da, respectively; carbamidomethyl (C) was included as a fixed modification; oxidation (M) and N-terminal protein acetylation were included as variable modifications; enzyme specificity was trypsin with up to two missed cleavages; and Arabidopsis sequences in the Swiss-Prot database (February 2017 release, 39,229 Arabidopsis sequence entries) were searched.

### *In Silico* Protein Digestions via MS-Digest

*In silico* proteolytic digestions of identified proteins – designed to generate peptide ions theoretically capable of being selected for MS/MS and identified via sequence database searches – were performed via MS-Digest (ProteinProspector version 5.19.4). MS-Digest was run using the following parameters: enzyme specificity was trypsin with up to two missed cleavages; peptide length was > 5 amino acid residues; and oxidation (M) and carbamidomethyl (C) were included as variable modifications. Theoretical peptide masses were used to calculate *m/z* values 350–2000 associated with peptide ions of charge state 2–5.

## Results

The efficacy of TDA/DDA should generally be greater than DDA for hypothesis driven data collection, but is more difficult to predict for non-hypothesis driven data collection. To investigate this we analyzed sample 1 using each TDA/DDA method and the DDA control method (*vide supra*). This allowed the effects of different TDA/DDA experimental designs to be studied. Sample 2 was analyzed using TDA_7_/DDA_7_ (method 3) and the DDA control method only to study specific effects relating to peptide quantification using ^15^N-labeled peptides (through comparisons to the equivalent TDA_7_/DDA_7_ (method 3) and DDA control method experiments conducted on the reciprocally labeled sample 1).

In presenting the results of these experiments, the average total quantities of MS/MS data collected using each TDA/DDA and DDA method are described first. Following this, the efficacies of the hypothesis driven and non-hypothesis driven components of TDA/DDA are presented separately. These efficacies are evaluated in relation to the three protein quantification steps illustrated in **Figure 1A**, with the criteria being: (1) the number of peptides and proteins identified following sequence database searching; (2) the number of heavy/light peptide pairs, and the number of proteins quantified using two or more such peptide pairs, following Proteome Discoverer analysis; and (3) the number of proteins quantified in this manner in three technical replicate experiments, and thus meeting the requirements for statistical significance testing as specified by Ting et al. (2009).

### Batch Effects

To ensure that the present results relate to differences between the TDA/DDA and DDA methods being investigated, and not differences in peptide ionization across experimental batches, analysis of potential batch effects was undertaken. Supplementary Figure S1 illustrates results showing the reproducibility of peptide ionization across LC-MS/MS batches 1.1–1.4 and 2.1–2.2 of **Figure 1C**. These results are described in full in the *Supplementary Material*. These results show reproducible peptide ionization efficiencies across the batches of experiments conducted on sample 1 (i.e., that peptide ion intensities remain consistent); however, significantly higher numbers of total MS signals in batch 1.4 (TDA_5_/DDA_5_ (method 2) experiments) were observed relative to batch 1.1 (control method experiments). For sample 2, a general decrease in the efficiency of peptide ionization in batch 2.2 (TDA_7_/DDA_7_ (method 3) experiments) was observed relative to batch 2.1 (control method experiments). The likely impacts of these batch effects are discussed in full in the Supplementary Material, and are taken into consideration in the reporting of results below.

### Comparative Quantity of MS/MS Events between TDA/DDA and DDA

**FIGURE 2:**
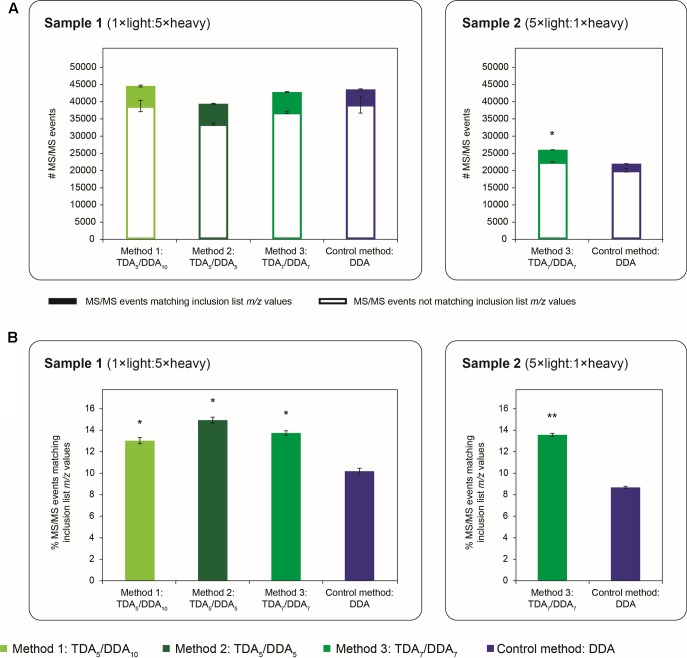
Quantity of MS/MS events obtained in TDA/DDA and DDA control method experiments. **(A)** Average numbers of MS/MS events per technical replicate in each batch of LC-MS/MS experiments conducted on sample 1 (left) and sample 2 (right); MS/MS events matching inclusion list *m/z* values are differentiated from those that do not; standard errors are shown for these two categories of MS/MS events. **(B)** Average percentages of MS/MS events matching inclusion list *m/z* values per technical replicate in each batch of LC-MS/MS experiments conducted on sample 1 (left) and sample 2 (right). Statistically significant differences between TDA/DDA and DDA control method experiments, as determined using 2-tailed *t*-tests, are labeled ^∗^*p* < 0.05 or ^∗∗^*p* < 0.01.

Previous studies have shown that, when using DDA alone, the total quantity of MS/MS data collected from an LC-MS/MS experiment can be dependent on the number of MS/MS events allocated to each scan cycle (Kalli and Hess, 2012; Kalli et al., 2013). **Figure 2** expands upon these studies by exploring this phenomenon when DDA is combined with TDA. In particular **Figure 2A** explores whether or not the addition of TDA prior to DDA in each scan cycle compromises the total quantity of MS/MS data collected, and **Figure 2B** explores the extent to which the use of TDA improves the selection of targeted *m/z* values. As the DDA control method allocates up to 10 MS/MS events per scan cycle, the 3 TDA/DDA methods studied here allow these questions to be explored for the following scenarios: maintenance of the same maximum number of DDA events per scan cycle as the control method while adding TDA events, as per TDA_5_/DDA_10_ (method 1); maintenance of the same maximum total number of MS/MS events per scan cycle as the control method by replacing DDA events with TDA events, as per TDA_5_/DDA_5_ (method 2); use of an intermediate maximum number of DDA events per scan cycle (relative to methods 1 and 2), while adding a high maximum number of TDA events per scan cycle (relative to methods 1 and 2), as per TDA_7_/DDA_7_ (method 3).

**Figure 2A** shows the average quantity of MS/MS data collected using each TDA/DDA and DDA method; MS/MS events which can be matched to inclusion list *m/z* values are shown separately from those that cannot. In sample 1 experiments, there are no significant differences in the average number of MS/MS events triggered using each method, or in the number of MS/MS events that cannot be matched to inclusion list *m/z* values. In contrast in sample 2, TDA_7_/DDA_7_ (method 3) experiments produce a significantly higher average number of MS/MS events (26,025 **±** 86 versus 21,982 **±** 570 for DDA; *p* = 0.014), and MS/MS events not matching inclusion list *m/z* values (22,494 **±** 74 versus 20,075 **±** 533 for DDA; *p* = 0.038) relative to the DDA control method. The relative differences between the TDA_7_/DDA_7_ (method 3) results across samples 1 and 2 can likely be attributed to the substantially different peptide abundances and compositions of these samples, as evidenced by the large differences in the total numbers of MS/MS events triggered from each sample (elaborated upon below). Nonetheless when taken together these results indicate that, for the samples studied here, the addition of 5–7 TDA events per scan cycle prior to DDA does not compromise the total amount of MS/MS data collected relative to the DDA control method.

**Figure 2B** shows the average percentages of MS/MS events that can be matched to inclusion list *m/z* values for each TDA/DDA and DDA method. Unsurprisingly, relative to the DDA control method, each TDA/DDA method produces a significant increase in the selection of inclusion list *m/z* values for MS/MS experiments (*p* = 0.012 for TDA_5_/DDA_10_ (method 1), *p* = 0.012 for TDA_5_/DDA_5_ (method 2) and *p* = 0.018 for TDA_7_/DDA_7_ (method 3) experiments conducted on sample 1; and *p* = 2.3 × 10**^-^**^3^ for the TDA_7_/DDA_7_ (method 3) experiments conducted on sample 2). No significant differences are observed between the use of 5 and 7 TDA events per scan cycle.

### Comparative Efficacies of Hypothesis Driven Data Collection between TDA/DDA and DDA

**FIGURE 3:**
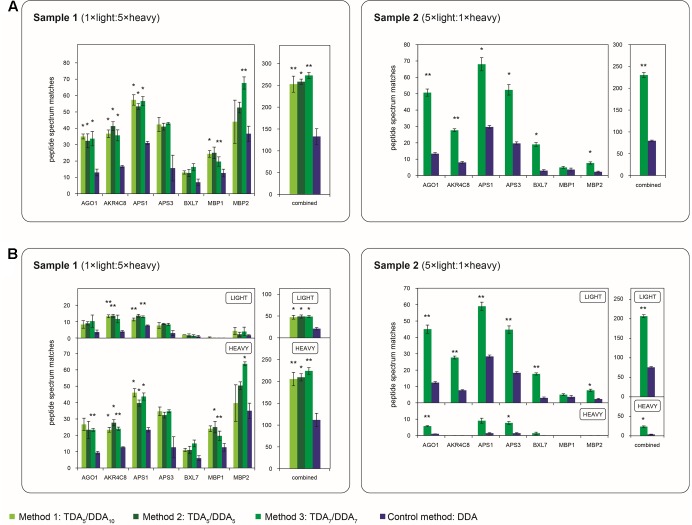
Average numbers of peptide spectrum matches obtained for the miRNA target proteins of **Table 1** in TDA/DDA and DDA control method experiments. **(A)** Average peptide spectrum matches for each individual targeted protein, and for all targeted proteins combined, per technical replicate in each batch of LC-MS/MS experiments conducted on sample 1 (left) and sample 2 (right). **(B)** The same data shown separately for light (above) and heavy (below) peptide spectrum matches. Statistically significant differences between TDA/DDA and DDA control method experiments, as determined using 2-tailed *t*-tests, are labeled ^∗^*p* < 0.05 or ^∗∗^*p* < 0.01.

**FIGURE 4:**
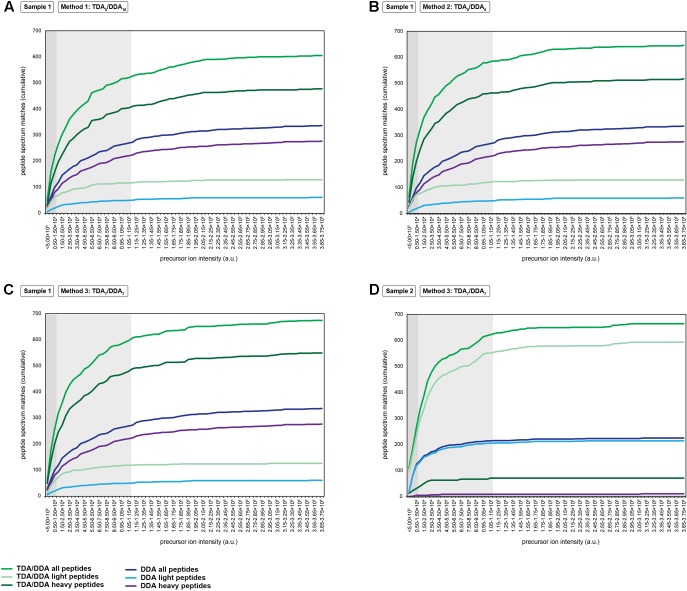
Cumulative precursor ion intensity distributions for peptide spectrum matches derived from the miRNA target proteins of **Table 1**. **(A)** Data from TDA_5_/DDA_10_ (method 1) experiments conducted on sample 1. **(B)** Data from TDA_5_/DDA_5_ (method 2) experiments conducted on sample 1. **(C)** Data from TDA_7_/DDA_7_ (method 3) experiments conducted on sample 1. **(D)** Data from TDA_7_/DDA_7_ (method 3) experiments conducted on sample 2. Panels **(A–C)** include data from DDA control method experiments conducted on sample 1, and panel D includes data from DDA control method experiments conducted on sample 2. All data are from combined sets of technical replicate TDA/DDA and DDA control method experiments. Moderate and low precursor ion intensities (respectively, defined as <1.50 × 10^4^ a.u. and <1.15 × 10^5^ a.u.) are highlighted in light gray and dark gray, respectively.

**FIGURE 5:**
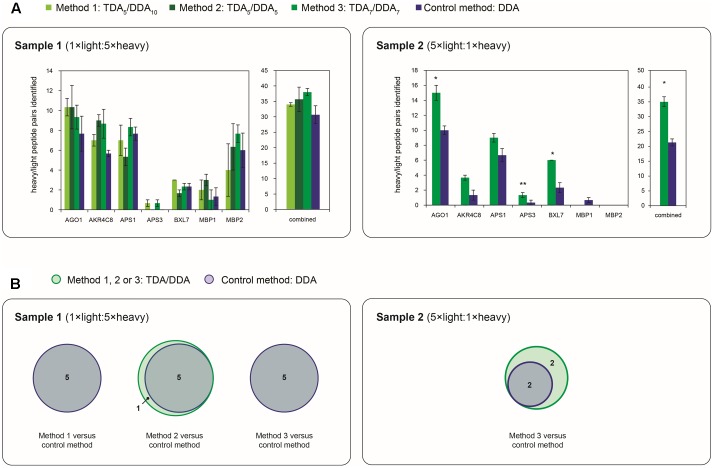
Depth and reproducibility of relative quantification of the miRNA target proteins of **Table 1** in TDA/DDA and DDA control method experiments. **(A)** Average numbers of heavy/light peptide pairs detected and quantified for each individual targeted protein, and for all targeted proteins combined, per technical replicate in each batch of LC-MS/MS experiments conducted on sample 1 (left) and sample 2 (right). Statistically significant differences between TDA/DDA and DDA control method experiments, as determined using 2-tailed *t*-tests, are labeled ^∗^*p* < 0.05 or ^∗∗^*p* < 0.01. **(B)** Numbers of targeted proteins quantified (from ≥ 2 heavy/light peptide pairs) across all three sets of technical replicates in TDA/DDA and DDA control method experiments conducted on sample 1 (left) and sample 2 (right).

To evaluate the efficacies of hypothesis driven data collection using the TDA/DDA methods relative to the DDA control method, results pertaining to the miRNA target proteins of **Table 1** are presented in **Figures 3–5**.

The performance of each method for step (1) in the determination of relative protein abundance levels using ^15^N-labeling – the identification of peptides and proteins from MS/MS data – is shown in **Figure 3**. **Figure 3A** shows the average number of peptide spectrum matches obtained for the targeted proteins per TDA/DDA or DDA experiment. This figure reveals that statistically significant increases in peptide spectrum matches are observed for 4, 3 and 5 of the 7 targeted proteins in the respective TDA_5_/DDA_10_ (method 1), TDA_5_/DDA_5_ (method 2) and TDA_7_/DDA_7_ (method 3) experiments conducted on sample 1, and 6 of the 7 targeted proteins in the TDA_7_/DDA_7_ (method 3) experiments conducted on sample 2. In all other instances targeted proteins are identified with higher average numbers of peptide spectrum matches in TDA/DDA methods relative to the DDA control method, but these increases are not statistically significant. When considering all of the targeted proteins together, all TDA/DDA experiments significantly outperform DDA control method experiments [*p* = 9.26 × 10**^-^**^5^ for TDA_5_/DDA_10_ (method 1), *p* = 0.019 for TDA_5_/DDA_5_ (method 2) and *p* = 6.8 × 10**^-^**^3^ for TDA_7_/DDA_7_ (method 3) experiments conducted on sample 1; and *p* = 1.1 × 10**^-^**^3^ for the TDA_7_/DDA_7_ (method 3) experiments conducted on sample 2].

**Figure 3B** illustrates the performance of each TDA/DDA and DDA method for the identification of light and heavy peptides separately. For all experiments conducted on sample 1, heavy peptide spectrum matches exceed light peptide spectrum matches, with the opposite being true of experiments conducted on sample 2. This is an expected consequence of the 5:1 heavy to light (sample 1) and light to heavy (sample 2) protein extract mixing ratios used during sample preparation. Relative standard errors are therefore generally high when considering the number of light peptide spectrum matches observed in sample 1 experiments, and when considering the number of heavy peptide spectrum matches observed in sample 2 experiments. As a result, in TDA/DDA experiments conducted on sample 1, more targeted proteins are identified with significant increases in heavy peptide spectrum matches than are identified with significant increases in light peptide spectrum matches relative to DDA control method experiments, with the opposite being true of sample 2. Nonetheless when all heavy or light peptide spectrum matches for targeted proteins are considered together, all TDA/DDA methods significantly outperform the DDA control method. This indicates that the use of TDA successfully increases the selection and identification of both unlabeled and ^15^N-labeled peptides.

A key aim of TDA is to improve the chances of identifying low abundance peptide ions that are bypassed for MS/MS when using DDA. **Figure 4** shows the relative abundances of the precursor ions from which the peptide spectrum matches of **Figure 3** are derived. It can be seen that, in sample 1, each TDA/DDA method most substantially outperforms the DDA control method in the identification of low abundance peptide ions (<1.50 × 10^4^ a.u.); this is true for both light and heavy peptides. In sample 2 this observation is even more pronounced; TDA/DDA experiments most substantially outperform the DDA control method experiments in the identification of the lowest abundance pool of peptide ions (<5.00 × 10^3^ a.u.) capable of being detected. Moreover it is evident that for both samples 1 and 2, each TDA/DDA method also produces an increase in the number of targeted peptide identifications derived from precursor ions of moderate intensity (<1.15 × 10^5^ a.u.) relative to the DDA control method.

The performance of each TDA/DDA method relative to the DDA control method for steps (2) and (3) in the determination of relative protein abundance levels using ^15^N-labeling – relative peptide quantification, and statistical significance testing – is illustrated in **Figures 5** and Supplementary Figure S3 for each targeted protein. Specifically **Figure 5A** shows the number of heavy/light peptide pairs detected and quantified from the TDA/DDA method and DDA control method experiments using Proteome Discoverer; the resulting heavy/light ratio measurements for these proteins are summarized in Supplementary Figure S3 and elaborated upon in the Supplementary Material. A comparison between **Figure 5A** and **Figure 3A** reveals that for each batch of experiments, substantially fewer heavy/light pairs are detected for targeted proteins compared to the number of peptides identified. This is unsurprising for two main reasons. Firstly, given that the majority of peptide spectrum matches for targeted proteins are derived from low abundance ions, as revealed in **Figure 4**, it can be expected that the isotopomer distributions of the partner peptides will often be below the detection limits of the employed orbitrap mass analyzer. Secondly, it is known that satellite peaks from the ^14^N-containing isotopomers of ^15^N-labeled peptides can reduce the efficiency of automated detections of heavy/light peptide pairs in metabolic ^15^N-labeling experiments (Arsova et al., 2012a).

**Figure 5A** reveals that, in sample 1 experiments, although average total numbers of heavy/light pairs detected using each TDA/DDA method are consistently higher than those detected using the DDA control method, these increases are not statistically significant. These results are in contrast to those obtained from sample 2. For each of the five proteins from which reliable heavy/light peptide pairs are detected in sample 2, TDA_7_/DDA_7_ (method 3) experiments led to the detection and quantification of a higher average number of heavy/light peptide pairs than DDA control method experiments, although these increases do not consistently alter the variability in heavy/light peptide ratio measurements (as indicated in Supplementary Figure S3). These increases are statistically significant for the targeted proteins AGO1, APS3 and BXL7. When considering all of the targeted proteins together, TDA_7_/DDA_7_ (method 3) significantly outperforms the DDA control method (35 ± 3 versus 21 ± 2 heavy/light peptide pairs detected, respectively; *p* = 0.038). It is possible that these improvements would have been even more pronounced were it not for the decreased efficiency of peptide ionization in TDA_7_/DDA_7_ (method 3) experiments relative to controls, as summarized in the section “Batch Effects.” **Figure 2** shows that, relative to sample 2, sample 1 contains substantially more peptide features that trigger MS/MS events matching those of targeted peptides, while **Figure 4** shows that these targeted peptides are generally identified from precursor ions of higher intensity than those of sample 2. This suggests that, in sample 2, TDA/DDA has significantly outperformed DDA when detecting extremely low abundance heavy/light peptide pairs for targeted proteins, while in sample 1, higher abundances of targeted proteins have resulted in less pronounced differences between TDA/DDA and DDA.

In considering the final step in the determination of relative protein abundance levels using ^15^N-labeling – step (3): downstream statistical significance testing – **Figure 5B** reveals that the differences between TDA/DDA and DDA are not pronounced in sample 1. This is because the DDA control method performs well on this sample; 5 of the 7 targeted proteins meet the criteria required for downstream statistical significance testing when using this method (i.e., they are quantified from 2 or more heavy/light peptide pairs in all three technical replicate LC-MS/MS experiments). This is in contrast to experiments conducted on sample 2, where TDA_7_/DDA_7_ (method 3) again outperforms the DDA control method. Specifically 4 targeted proteins (AGO1, AKR4C8, APS1, and BXL7) meet the criteria required for such downstream statistical significance testing in the TDA_7_/DDA_7_ (method 3) experiments. In comparison only 2 of the targeted proteins (AGO1 and APS1) meet these criteria in the DDA control method experiments. Moreover, the statistical significance tests conducted on AGO1 and APS1 carry more power in the TDA_7_/DDA_7_ (method 3) experiments when compared to the DDA control method experiments. This is because these proteins are quantified from a larger sample size of heavy/light peptide pairs, as described above.

### Comparative Efficacies of Non-hypothesis Driven Data Collection between TDA/DDA and DDA

**FIGURE 6:**
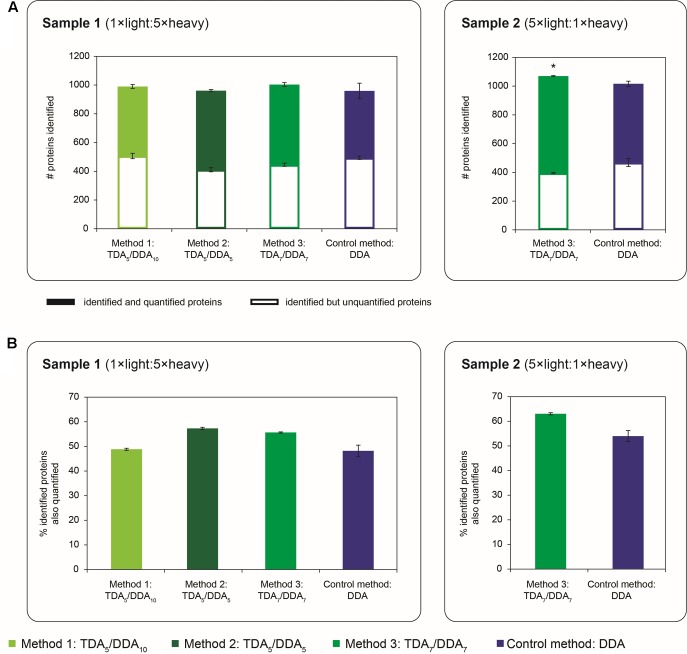
Quantity of proteins identified and quantified in TDA/DDA and DDA control method experiments. **(A)** Average numbers of proteins identified (from ≥ 2 significantly scoring peptide spectrum matches) per technical replicate in each batch of LC-MS/MS experiments conducted on sample 1 (left) and sample 2 (right); identified and quantified proteins are differentiated from proteins that are identified but not quantified; standard errors are shown for these two categories of identified protein. **(B)** Average percentages of identified proteins that are also quantified (from ≥ 2 heavy/light peptide pairs) per technical replicate in each batch of LC-MS/MS experiments conducted on sample 1 (left) and sample 2 (right). Statistically significant differences between TDA/DDA and DDA control method experiments, as determined using 2-tailed *t*-tests, are labeled ^∗^*p* < 0.05.

**FIGURE 7:**
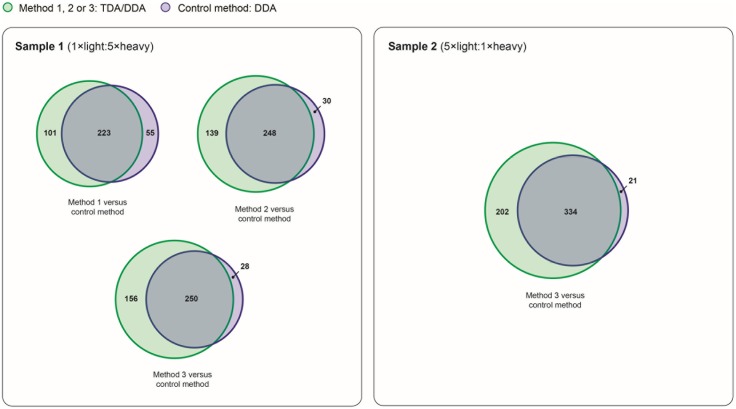
Reproducibility of relative protein quantification in TDA/DDA and DDA control method experiments. Numbers of proteins quantified (from ≥ 2 heavy/light peptide pairs) across all three sets of technical replicates in TDA/DDA and DDA control method experiments conducted on sample 1 (left) and sample 2 (right).

To evaluate the efficacies of non-hypothesis driven data collection using each TDA/DDA method relative to the DDA control method, results pertaining to the broad-scale identification of Arabidopsis proteins are presented in **Figures 6**, **7**.

The broad-scale performance of each method for step (1) in the determination of relative protein abundance levels using ^15^N-labeling – the identification of peptides and proteins from MS/MS data – is shown in **Figure 6A**. Specifically **Figure 6A** shows the average number of Arabidopsis proteins identified from 2 or more peptide spectrum matches per TDA/DDA or DDA experiment (990 ± 31 in TDA_5_/DDA_10_ (method 1), 961 ± 23 in TDA_5_/DDA_5_ (method 2), 1004 ± 24 in TDA_7_/DDA_7_ (method 3) and 959 ± 66 in DDA control method experiments conducted on sample 1; and 1070 ± 2 in TDA_7_/DDA_7_ (method 3) and 1016 ± 12 in DDA control method experiments conducted on sample 2). When considering the results obtained from samples 1 and 2 separately, these results mirror the results shown in **Figure 2**; i.e., for each sample, the relative quantity of MS/MS data collected using each method dictates the number of proteins identified. Importantly these results confirm that the addition of 5–7 TDA events per scan cycle prior to DDA does not compromise the collection of non-hypothesis driven data relative to the DDA control method. Moreover these results show that, for the samples studied here, the quantity of non-hypothesis driven data that is collected is not significantly affected by the number of DDA events per scan cycle when between 5 and 10 DDA events are used.

Interestingly **Figure 6A** also reveals that experiments conducted on sample 2 identify a similar number of Arabidopsis proteins to experiments conducted on sample 1. This is despite the fact that sample 2 triggers fewer MS/MS events and has generally lower peptide abundances than sample 1, as discussed previously. To explain this observation, it should be noted that the monoisotopic peaks of ^15^N-labeled peptides can be difficult to select for MS/MS due to satellite peaks from ^14^N-containing isotopomers; this negatively impacts upon the number of heavy peptides identified in ^15^N-labeling experiments (Arsova et al., 2012b). As sample 1 has a substantially higher proportion of ^15^N-labeled peptides than sample 2, it is therefore unsurprising that this sample produces a proportionally lower number of protein identifications relative to the amount of MS/MS data collected.

The broad-scale performance of each TDA/DDA method relative to the DDA control method for step (2) in the determination of relative protein abundance levels using ^15^N-labeling – relative peptide quantification – is also illustrated in **Figure 6**. As with the observations made for the hypothesis driven data, **Figure 6A** shows that each TDA/DDA and DDA method unsurprisingly identifies more proteins than it quantifies (when quantification is performed using 2 or more heavy/light peptide pairs). **Figure 6B** shows that, for each sample, the relative proportions of identified proteins that are also quantified do not differ significantly between each TDA/DDA method and the DDA control method. Taken together these results reinforce the finding that, relative to the DDA control method, the TDA/DDA methods studied here are not compromised in their ability to collect non-hypothesis driven quantitative proteomics data.

The broad-scale performance of each TDA/DDA method and the DDA control method for step (3) in the determination of relative protein abundance levels using ^15^N-labeling – downstream statistical significance testing – is illustrated in **Figure 7**. Interestingly each TDA/DDA method produces substantially more proteins that meet the criteria required for downstream statistical significance testing than the DDA control method, despite the fact that TDA inclusion lists were specifically tailored to the miRNA target proteins of **Table 1**. This is true for both samples 1 and 2. When considering the 4 comparisons between the TDA/DDA methods and the DDA control method across samples 1 and 2, a significantly higher percentage of proteins uniquely meet downstream statistical significance testing criteria in TDA/DDA experiments than in DDA experiments (33.0 ± 2.6% versus 8.0 ± 2.7%, respectively; *p* = 0.011 as determined using a 2-tailed *t*-test).

When considering this unexpected result it is notable that, in sample 2, TDA_7_/DDA_7_ (method 3) experiments produce a significant ∼7% average increase in total MS/MS events relative to DDA control method experiments, as discussed in relation to **Figure 2**. These differences in total MS/MS events may have produced some additional identifications of non-targeted peptides when using TDA/DDA relative to DDA (i.e., a maximum increase of ∼7%); however, they cannot account for the ∼51% increase in quantified peptides shown in **Figure 7**. Moreover **Figure 2** shows that, for experiments conducted on sample 1, none of the TDA/DDA methods produce a significantly higher average number of MS/MS events than the DDA control method.

Taken together, the above considerations suggest that peptides from non-targeted proteins with *m/z* values matching those in the inclusion lists, within the ± 10 ppm mass measurement errors utilized in the present methods, are consistently being selected for MS/MS using TDA. This hypothesis is reinforced by Supplementary Figure S2 which shows that, in sample 1 experiments, there is a large pool of proteins that are commonly identified (78 in total) and quantified (58 in total) across each set of technical replicate TDA/DDA experiments, but not in DDA control method experiments. Further reinforcement of this hypothesis is found by comparing inclusion list *m/z* values to *m/z* values theoretically capable of being produced from non-targeted proteins in the Arabidopsis proteome. Specifically *in silico* digestion the 1064 non-targeted proteins identified from > 1 unique peptide in sample 2 generates 1,061,263 theoretical light peptide ions (when following the *in silico* digestion procedures detailed in the section “Materials and Methods”). Of these peptide ions, 37,106 (3.5%) have *m/z* values matching those of the light peptide ions in the present inclusion lists (within a mass measurement error of ± 10 ppm). This confirms that considerable redundancy in *m/z* values can be expected when analyzing complex peptide mixtures derived from large pools of proteins. It it therefore unsurprising that the present inclusion lists, which utilized open retention time windows, may have resulted in the repeated selection of peptides from non-targeted proteins for MS/MS. These findings are elaborated upon in the section “Discussion.”

## Discussion

### TDA/DDA Outperforms DDA for Both Hypothesis Driven and Non-hypothesis Driven Data Collection

This study has provided the first in-depth investigation into the performances of TDA and DDA when they are used in combination. Specifically it has provided new insights into the efficacy of this MS/MS data acquisition strategy toward relative protein quantification, using mixed unlabeled and ^15^N-labeled peptide samples of a typical complexity for a quantitative plant proteomics experiment.

The results described here demonstrate that the present TDA/DDA methods consistently outperform the DDA control method in the selection and identification of targeted peptides. Of particular interest is the fact the use of inclusion lists during TDA/DDA improves the identification of heavy peptides from targeted proteins. This overcomes a specific shortcoming of the metabolic ^15^N-labeling strategy, the inefficient selection of ^15^N-labeled peptides for MS/MS identification (Arsova et al., 2012b). These increases in targeted peptide identifications result in significant increases in the detection of associated heavy/light peptide pairs when particularly low abundance samples of targeted proteins are analyzed; i.e., when targeted proteins are particularly difficult to identify and quantify using DDA alone, as observed in sample 2.

It is also demonstrated that these improvements in the hypothesis driven selection and identification of peptides when using TDA/DDA do not compromise the collection of broad-scale non-hypothesis driven data. This is evidenced by the fact that the TDA/DDA methods studied here identify and quantify as many proteins as the DDA control method, or in the case of the TDA_7_/DDA_7_ (method 3) experiments conducted on sample 2, significantly more proteins.

Importantly, for the datasets described here, TDA/DDA is also demonstrated to be capable of outperforming DDA when considering the third and final step of protein relative quantification when using the metabolic ^15^N-labeling strategy: the downstream statistical analysis of protein expression levels. When considering hypothesis driven data collection only, these outcomes are observed in sample 2; they are a logical consequence of the improved performance of TDA/DDA relative to DDA in the identification and relative quantification of targeted peptides in this sample. However, these results also indicate that, even when the overall efficacy of relative peptide quantification is not significantly different between the two methods, TDA/DDA can still outperform DDA during downstream statistical significance testing for individual proteins. For example, relative to DDA control method experiments, the TDA/DDA experiments conducted on sample 2 did not produce significantly more detected and quantified heavy/light peptide pairs for AKR4C8 (**Figure 5A**). However, AKR4C8 was quantified using multiple heavy/light peptide pairs in all 3 TDA/DDA experiments, compared to only 2 of 3 DDA control method experiments. This suggests that, when considering the criteria for downstream statistical significance testing, inclusion lists can be advantageous because they lead to increased reproducibility of targeted peptide quantification, regardless of whether or not they also increase the overall number of targeted peptides that are quantified.

The above reasoning can also explain the unexpected improvements in non-hypothesis driven data collection observed in **Figure 7**. Specifically, relative to the DDA control method, the number of quantified proteins shown in **Figure 7** is disproportionately high for each TDA/DDA method when considering the quantity of MS/MS data collected using each method. This suggests the following: the inclusion lists utilized in this study (which targeted a total of 1558 distinct *m/z* values; see **Table 1**) unintentionally lead to the selection of a substantial number of non-targeted peptides for MS/MS via TDA; these particular peptides are reproducibly selected for MS/MS via TDA across technical replicate experiments; and that these particular peptides are generally bypassed for MS/MS when using DDA. This hypothesis is consistent with the fact that complex peptide samples were subjected to LC-MS/MS analysis (between 32 and 61k peptide features were observed per LC-MS/MS experiment), and that considerable redundancy in targeted and non-targeted peptide ion *m/z* ratios can be expected in these samples (*vide supra*). It is therefore unsurprising that some non-targeted peptides produced *m/z* values matching those in inclusion lists. This hypothesis is also consistent with findings reported by Savitski et al. (2010), who demonstrated that TDA enhances the reproducibility of peptide identifications relative to DDA. These observations therefore suggest that if targeted peptide inclusion list sizes are increased relative to those utilized in this study, the number of non-targeted proteins that are reproducibly quantified across experiments may also further increase.

### Considerations in the Design and Broad-Scale Applicability of TDA/DDA Experiments

The samples analyzed in this study are associated with a specific metabolic ^15^N-labeling experiment, conducted on an LTQ Orbitrap Velos Pro instrument platform. The degree to which TDA/DDA may offer improvements over DDA in any other given quantitative proteomics experiment will be dictated by a variety of factors. Examples of these factors include, but are not limited to: the employed protein quantification strategy; the abundance and number of targeted peptides; overall sample abundance and complexity; and the duty cycle and mass analyzer detection limits of the employed mass spectrometric instrumentation. It can, however, be expected that the advantages of TDA/DDA over DDA observed in the present study will be broadly applicable across sample types and instrument platforms. This is because the potential for improved hypothesis driven data collection via TDA exists whenever DDA does not efficiently select peptide ions of interest for MS/MS (Schmidt et al., 2008; Domon et al., 2009; Savitski et al., 2010; Hart-Smith et al., 2012), while the present results also indicate that, if designed carefully, TDA/DDA will rarely compromise broad-scale data acquisition relative to DDA. These points are elaborated upon below.

Of the three TDA/DDA methods studied here, all perform similarly. When considering the quantity of hypothesis driven data collected using each TDA/DDA method, it is useful to note that each method allocates a maximum of either 33% (method 1) or 50% (methods 2 and 3) of its MS/MS events to TDA, but that TDA events will not be triggered if peptides with monoisotopic masses matching those of inclusion list *m/z* values cannot be detected. **Figure 2B** reveals that only between 13 and 15% of MS/MS events match inclusion list *m/z* values in experiments performed using the present TDA/DDA methods. This indicates that, on average, the number of TDA events triggered per scan cycle is substantially less than the maximum allocated 5–7, and that a large proportion of the inclusion list *m/z* values capable of being detected in these LC-MS/MS experiments have indeed been selected for MS/MS. When considering the quantity of non-hypothesis driven data collected using these methods, no significant differences are observed when allocating between 5 and 10 DDA events per scan cycle, with the possible exception of when 5 DDA events are allocated (for more details, see the section “Batch Effects” (extended analysis) of the Supplementary Material). Together these findings suggest that similar results would have been obtained from the present samples using any combination of TDA/DDA featuring an allocation of >15% of MS/MS events to TDA, and 5–10 DDA events per scan cycle, on the LTQ Orbitrap Velos Pro instrumentation employed here.

The above observations indicate that the use of TDA prior to DDA in LC-MS/MS scan cycles does not, in and of itself, negatively impact upon the total quantity of MS/MS data collected. When TDA events are triggered they either select targeted peptides, or serendipitously select untargeted peptides for MS/MS; both cases contribute to the overall quantity of MS/MS data collected. Therefore the quantity of MS/MS data collected from a given TDA/DDA experiment is likely to depend upon the average number of MS/MS events per scan cycle, as observed in previous studies conducted using DDA only (Kalli and Hess, 2012; Kalli et al., 2013), irrespective of whether or not these events are triggered by TDA or DDA. Kalli et al. (2013) have previously noted that, when using LTQ Orbitrap Elite instrument platforms, the quantity of MS/MS data collected is lowered when relatively few MS/MS events are triggered per scan cycle; a particularly high number of MS/MS events per scan cycle (15–20) does not compromise the quantity of data collected, but also offers no advantages. This would suggest that for samples of similar complexity to those studied here, TDA/DDA methods should be designed to allocate a similar number of DDA events per scan cycle as an optimized DDA only method, together with an excess number of allocated TDA events (e.g., > 5 for the present instrumentation when targeting 1558 *m/z* values). For studies with a high number of targeted proteins – e.g., with inclusion lists designed to target over an order of magnitude more *m/z* values than the 1558 used here – it is possible that the number of peptides capable of triggering TDA events will reach those of peptides capable of triggering DDA events. In such a scenario, if the total quantity of MS/MS data is to be maximized, the following experimental design should be utilized: allocation of the same number of TDA and DDA events to each scan cycle, with the same total number of MS/MS events per scan cycle as an optimized DDA only method.

Though the present study is focused on the metabolic ^15^N-labeling of plants, it is likely that alternative relative quantification strategies that are applicable to both plant and non-plant systems (e.g., stable isotope labeling by amino acids in cell culture (SILAC), chemical labeling and label-free) will also benefit from TDA/DDA. This is because, as with metabolic ^15^N-labeling, the depths of proteome coverage achieved using these other strategies are compromised by the shortcomings of DDA when standard LC-MS/MS methods are used for peptide identification and quantification (Li et al., 2012; Miller et al., 2013), or in the generation of DIA assay libraries (Schubert et al., 2015). The extents to which TDA/DDA methods carry the potential to outperform DDA methods when using these other strategies will depend on the specific quantification strategy used and the nature of the samples under study. However, for samples of similar proteome complexity to those studied here, it can be envisaged that quantification strategies that do not rely on the detection of heavy/light peptide pairs, and instead use individual or isobaric peptides for quantification, will particularly benefit from TDA/DDA. These include label-free strategies that make use of peptide intensities [e.g., MaxLFQ (Cox et al., 2014)] or spectral counting [e.g., the Exponentially Modified Protein Abundance Index (emPAI) (Ishihama et al., 2005)], or chemical labeling strategies that make use of isobaric mass tags. This is because in the ^15^N-labeling based quantification workflows studied here, TDA/DDA more consistently produces significant increases in peptide identifications – the primary determinant of the depth of label-free or isobaric mass tag-based quantification – than in heavy/light peptide pair detection. Moreover it can be envisaged that the strong reproducibility of TDA LC-MS/MS experiments, observed here and previously (Savitski et al., 2010), may particularly benefit spectral counting strategies. This is because the accuracies of these strategies are directly reliant on peptide ions of equivalent intensity being consistently selected for fragmentation across LC-MS/MS experiments. Peptide ions subjected to MS/MS in one TDA experiment and not another are likely to reflect a genuine decrease in ion intensity, rather than the stochastic nature of DDA.

The present results also indicate that TDA/DDA should be widely applicable to non-quantitative studies that aim to target specific proteins while collecting broad-scale data. We have, for example, recently applied TDA/DDA to the non-quantitative study of mammalian cholesterol synthesis enzymes (Luu et al., 2015). Future in-depth investigations into the performance of TDA/DDA relative to DDA in other experimental workflows may be therefore worthwhile.

## Conclusion

It can be envisaged that large-scale proteomic quantification techniques will play a crucial role in furthering our understanding of plant proteins. Both hypothesis driven and non-hypothesis driven LC-MS/MS data acquisition strategies offer powerful avenues by which such studies can be pursued. The present results demonstrate that TDA/DDA offers a means by which the advantages of both strategies can be kept without compromise. That is, TDA/DDA can significantly increase the depth of proteome coverage for targeted proteins, while collecting broad-scale quantification data for non-targeted proteins in a manner that is more reproducible than a purely non-hypothesis driven DDA approach.

Taken together these findings suggest that TDA/DDA should be considered for use in quantitative plant proteomics studies whenever it would be beneficial to quantify proteins in a broad-scale unbiased manner, while concomitantly targeting particular proteins of known or hypothesized biological interest for quantification. Moreover these findings suggest that TDA/DDA is a currently underutilized LC-MS/MS method, and that its efficacies in experimental workflows and sample types beyond those typical of quantitative plant proteomics are worthy of further investigation.

## Author Contributions

GH-S, RR, and MW conceived and designed the proteomics experiments. GH-S performed the mass spectrometry experiments and data analysis. RR and PW conceived all other experiments. RR performed all other experiments. GH-S wrote the manuscript. All authors read and approved the final manuscript.

## Conflict of Interest Statement

The authors declare that the research was conducted in the absence of any commercial or financial relationships that could be construed as a potential conflict of interest.
