# Supplementary material for: Improved Quantitative Plant Proteomics via the Combination of Targeted and Untargeted Data Acquisition
Source: Front Plant Sci. 2017 Sep 27;8:1669. doi: 10.3389/fpls.2017.01669 (PMC5623951; doi:10.3389/fpls.2017.01669)
Supplement: Supplementary file 1 [file Data_Sheet_1.docx]

**improved quantitative plant proteomicsvia the combination of targeted and untargeted data acquisition**

Gene Hart-Smith,^1^*Rodrigo Siqueira Reis,^2,3^Peter Waterhouse,^2,4^Marc R. Wilkins^1^

^1^NSW Systems Biology Initiative, School of Biotechnology and Biomolecular Sciences, University of New South Wales, Sydney, New South Wales 2052, Australia

^2^School of Biological Sciences, University of Sydney, Macleay Building A12, Sydney, NSW 2006, Australia

^3^Department of Plant Molecular Biology, University of Lausanne, Lausanne, CH-1015, Switzerland

^4^Centre for Tropical Crops and Biocommodities, Queensland University of Technology, Brisbane, QLD 4001, Australia

Corresponding author: Gene Hart-Smith (Postal address: Room 263 Biological Sciences Building, University of New South Wales, Sydney, New South Wales 2052, Australia; Phone: +61-2-9385-3633; Fax, +61-2-9385-3950; Email: g.hart-smith@unsw.edu.au)

Email addresses of other authors: Rodrigo Siquera Reis: Rodrigo.SiqueiraReis@unil.ch; Peter Waterhouse: peter.waterhouse@qut.edu.au; Marc R. Wilkins: m.wilkins@unsw.edu.au

**SUPPLEMENTARY MATERIAL**

**Batch effects (extended analysis)**

**
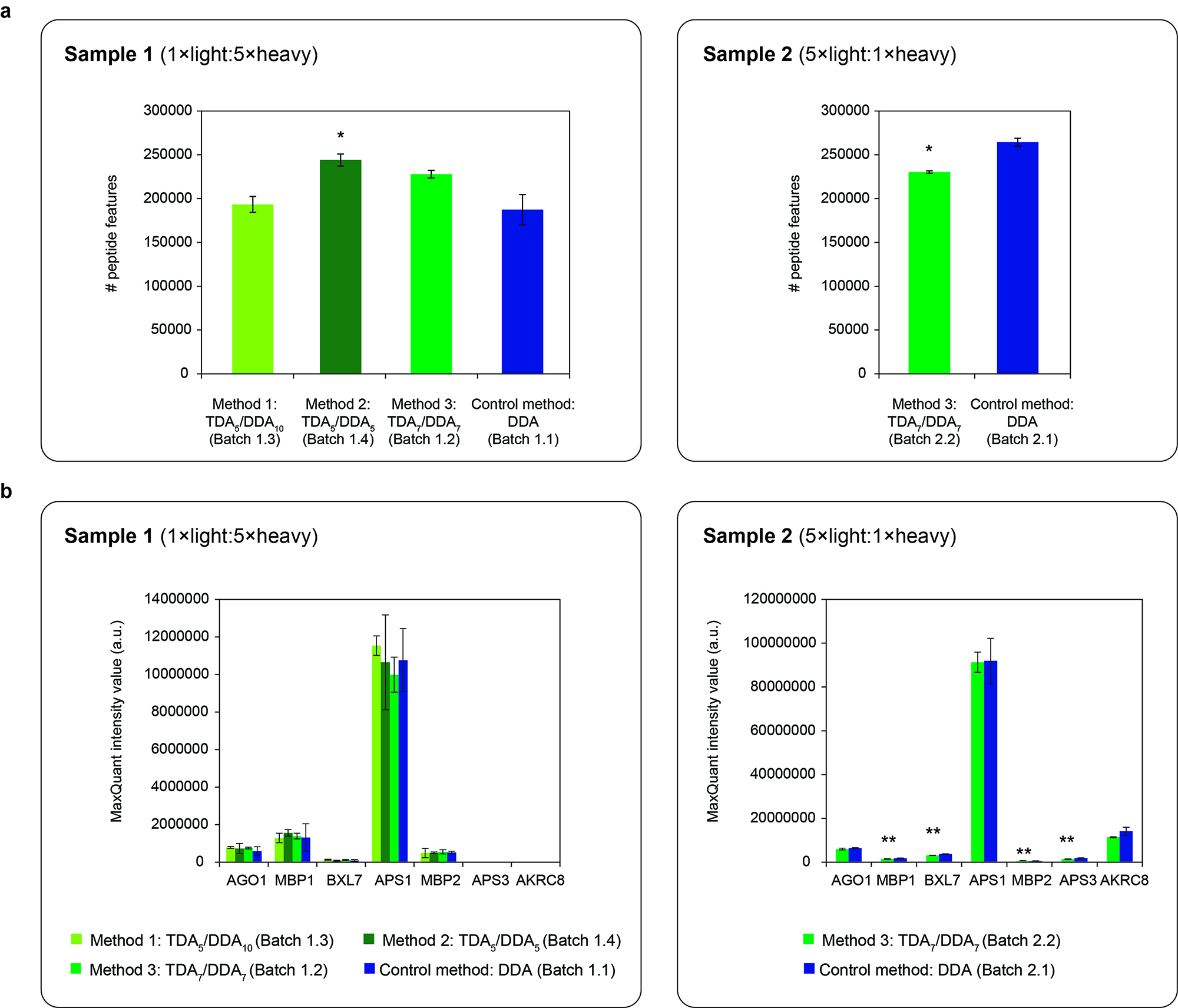
**

**Figure S1.** Measures of peptide ionisationefficiencyinthebatches of TDA/DDA and DDA experiments summarised in Figure 1c.**a** Average numbers of peptide features detected by MaxQuant per set of technical replicate LC-MS/MS experiments for sample 1 (left) and sample 2 (right). **b** Average protein signal intensities (i.e. summed peptide ion intensities) for the miRNA target proteins of Table 1per set of technical replicate LC-MS/MS experiments for sample 1 (left) and sample 2 (right), as measured by MaxQuant using light peptide ions only (as Andromeda is unable to identify ^15^N-labeled peptides in sequence database searches). Statistically significant differences between batches of TDA/DDA and DDA control method experiments, as determined using 2-tailed *t­*-tests, are labeled * (*p*<0.05) or ** (*p*<0.01).

Figure S1a shows, for each batch of experiments summarised in Figure 1c,the average number of peptide features detected by MaxQuantper set of technical replicate LC-MS/MS experiments.In sample 1, there is a significant (*p* = 0.044) increase in peptide features in batch 1.4(TDA_5_/DDA_5_ (method 2) experiments)relative to batch 1.1 (control method experiments). In sample 2, there is a significant (*p* = 0.027) decrease in peptide features in batch 2.2 (TDA_7_/DDA_7_ (method 3) experiments)relative to batch 2.1 (control method experiments). Other TDA/DDAbatches show no significant differences in peptide features relative to their respective DDA control method batches.

Figure S1b shows, for each batch, the averagelightprotein signal intensities for the miRNA target proteins subjected to TDA, as detected by MaxQuant. In sample 1, no significant batch effects are observed for any of these protein signal intensities. In sample 2, statistically significant (*p* < 0.01) decreases in signal intensity are observed for 3 proteins in batch 2.2 (TDA_7_/DDA_7_ (method 3) experiments) relative to batch 2.1 (control method experiments), while 1 protein, MBP2, showsa statistically significant (*p* = 1.1×10^-3^) increase.

Together these results indicate reproducible peptide ionisation efficiencies across the batches of experiments associated with sample 1. While peptide features apparently increase in batch 1.4 (TDA_5_/DDA_5_ (method 2) experiments),peptide ion intensitiesfor the targeted Arabidopsis proteins do not also increase. The additional signals in these MS data are therefore unlikely to be derived from Arabidopsis peptide ions, and may instead be derived from non-Arabidopsis or non-peptide contaminants. For sample 2, the results shown in Figure S1suggesta general decrease inthe efficiency of peptide ionisation in batch 2.2 (TDA_7_/DDA_7_ (method 3) experiments) relative to batch 2.1 (control method experiments).

*Likely impacts upon the comparative quantity of MS/MS events between TDA/DDA and DDA*

When considering Figure 2a and the TDA_5_/DDA_5_ (method 2) experiments conducted on sample 1, it is possible that a significantly lower average amount of MS/MS data would have been collected, relative to the DDA control method, were it not for the abovementioned batch effects. Kalli et al. previously reported that, when AGC target values and ion injection times are held constant across LC-MS/MS experiments on an LTQ Orbitrap Elite instrument platform, the implementation of fewer DDA events per scan cycle can produce fewer total MS/MS events (Kalli, Smith, Sweredoski and Hess 2013). This general finding can be expected to hold true for the LTQ Orbitrap Velos Pro instrument platform utilised here. As the present TDA_5_/DDA_5_ (method 2) experiments utilised 5 DDA events per scan cycle compared to 10 for the control method, a significant decrease in the amount of MS/MS data collected using this method would therefore not be surprising.

When considering the TDA_7_/DDA_7_ (method 2) experiments conducted on sample 2, it is possible that the already significantly increased amount of MS/MS data collected relative to the DDA control method would have been even higher were it not for the abovementioned batch effects.

Neither of the abovementioned scenarios impacts upon the primary implication drawn from the data shown in Figure 2a; that is, for the samples studied here, the addition of 5-7 TDA events prior to DDA is unlikely to compromise the total amount of MS/MS data that can be collected when using DDA alone.

*Likely impacts upon the comparative efficacies of hypothesis driven data collection between TDA/DDA and DDA*

Figure S1bindicates that for the TDA_7_/DDA_7_ (method 2) experiments conducted on sample 2, batch effects affected the intensities of the precursor ions from which the miRNA target proteins of Table 1 are identified. This impacts upon the precursor ion intensity data shown in Figure 4. Although these batch effects are significant, they are not large. The changes in average signal intensity for the 3 proteins showing significant decreases – MBP1, BXL7 and APS3 – are respectively -17%, -16% and -24% relative to DDA control method experiments. This suggests that batch effects are unlikely to have impacted upon the primary observation made from these data; i.e. that these experiments most substantially outperform the DDA control method experiments in the identification of particularly low abundance peptide ions.

**Comparative reproducibilities of protein identification and quantification in TDA/DDA and DDA experiments conducted on sample 1**

**
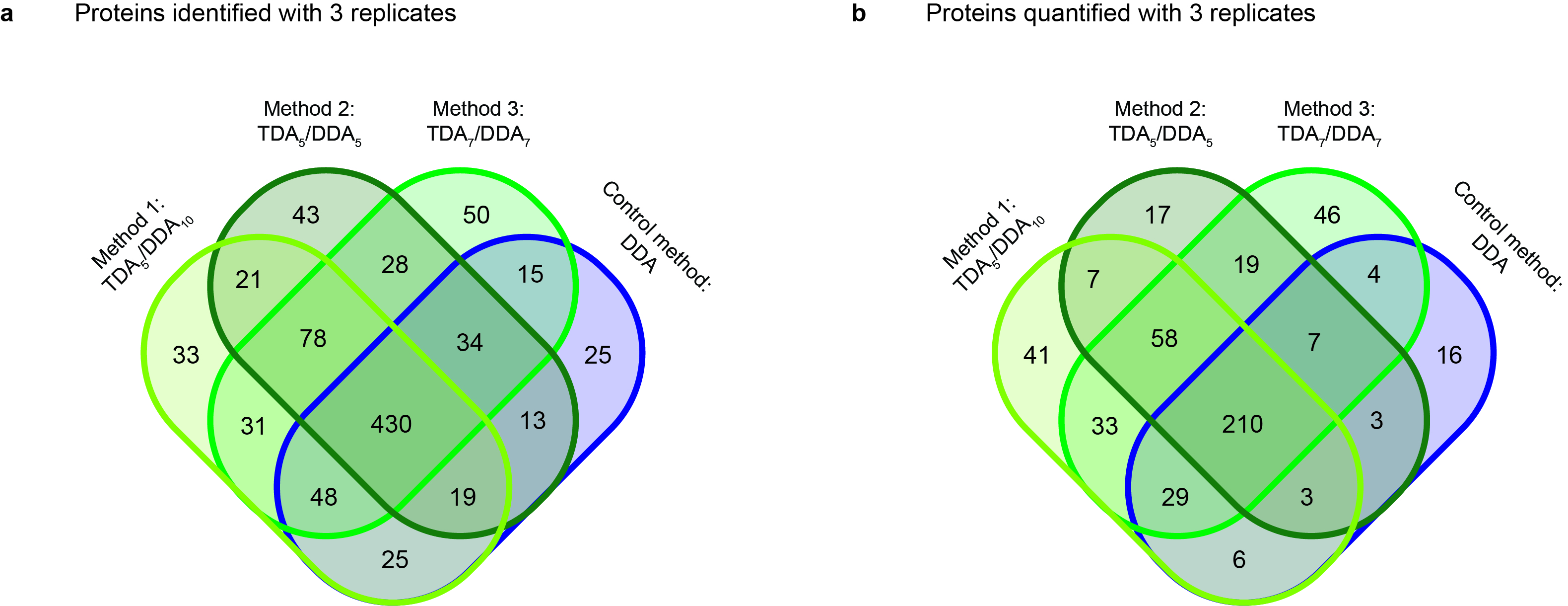
**

**Figure S2.** Reproducibilities of relative protein identification and quantification in all TDA/DDA and DDA control method experiments conducted on sample 1. **a** Numbers of proteins identified (from ≥2 significantly scoring peptide spectrum matches) across all 3 sets of technical replicatesin TDA/DDA and DDA control method experiments conducted on sample 1. **b** Numbers of proteins quantified (from ≥2 heavy/light peptide pairs) across all 3 sets of technical replicatesin TDA/DDA and DDA control method experiments conducted on sample 1.

**Heavy to light ratios ofmiRNA target proteins**

**
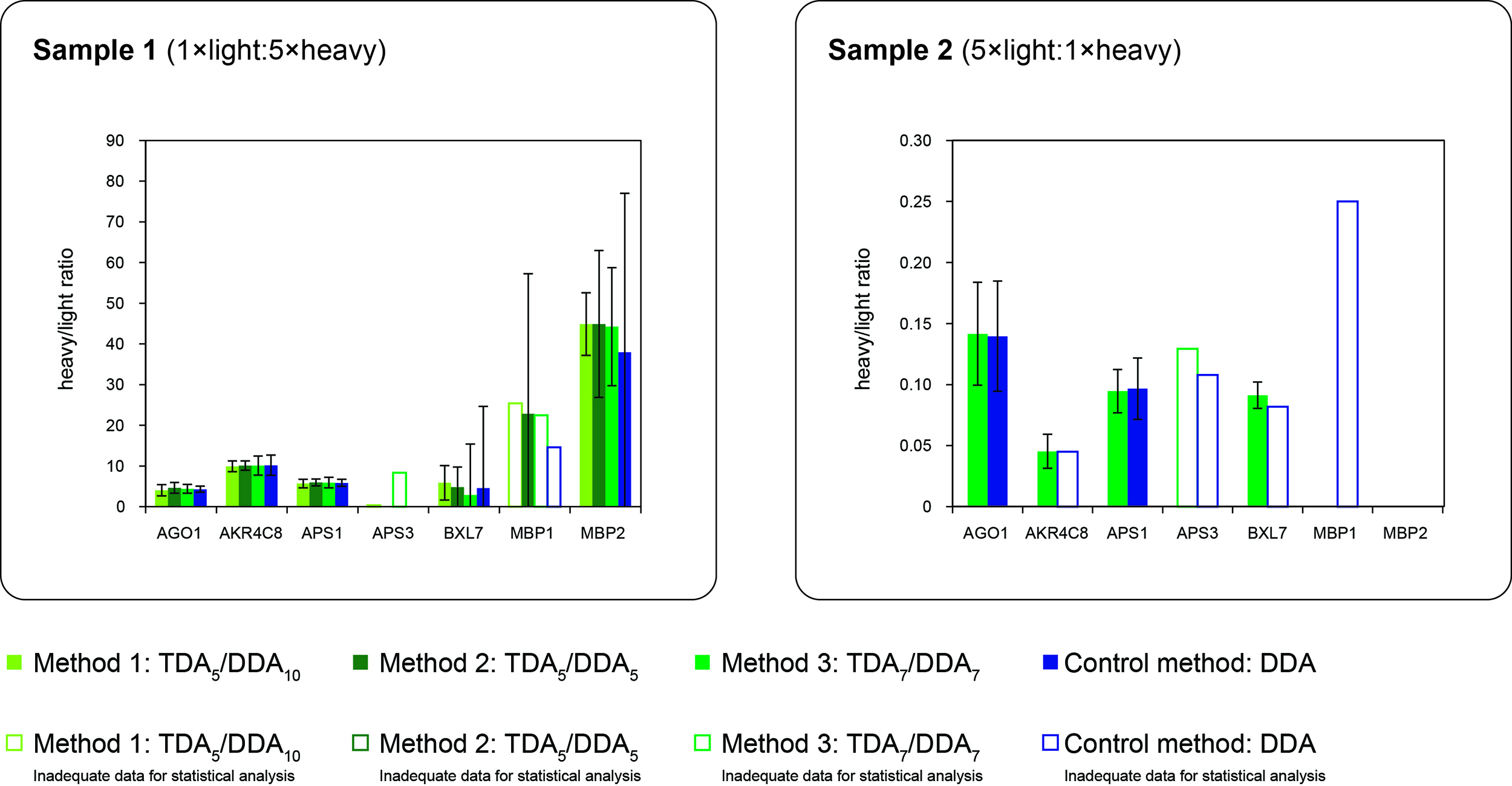
**

**Figure S3.** Heavy/light ratios for the miRNA target proteins of Table 1 in TDA/DDA and DDA control method experiments. Average heavy/light ratiosperset of technical replicate LC-MS/MS experimentsare shown for sample 1 (left) and sample 2 (right). Standard errors are estimated from data collected across 3 sets of technical replicates as specified by [Ting, et al. (2009](#_ENREF_38)); data from which standard errors cannot be estimated in this manner are shown in outlined boxes.

Figure S3illustrates heavy/light ratio measurements for the miRNA target proteins of Table 1 obtained from the present TDA/DDA and DDA control method experiments. As only a limited number of proteolytic peptide mixtures from samples 1 and 2 were subjected to LC-MS/MS analysis this study, broad-scale heavy/ratio normalisation and subsequent statistical analyses of protein fold-changes following[Ting, et al. (2009](#_ENREF_38))were not conducted here.In-depth analyses of protein accumulation in *drb1*relative to wild-type Arabidopsis are therefore not reported. Nonetheless the present data are consistent with our previous analyses of *drb1* versus wild-type Arabidopsis (Reis, et al. 2015). Specifically a median heavy (*drb1*) to light (wild-type) protein ratio of ~6.1 was observed for all proteins quantified from sample 1, while a median heavy (wild-type) to light (*drb1*) protein ratio of ~0.11 was observed for all proteins quantified from sample 2.Relative to these median values, the heavy/light ratios for AKR4C8, MBP1 and MBP2 shown in Figure S3 are consistent with our previous finding that these proteins over-accumulate in *drb1* relative to wild-type Arabidopsis (Reis, et al. 2015), when considering quantitative data obtained from 3 technical replicate experiments only (i.e. only the filled boxes with error bars in Figure S3). Similarly the present data are consistent with under-accumulation of AGO1, and non-significant differences in APS1, APS3 and BXL7 abundance in *drb1* relative to wild-type Arabidopsis, as reported previously (Reis, et al. 2015).

Figure S3 indicates that heavy/light ratio standard errors (estimated from the variability of peptide-level heavy/ratios) observed in TDA/DDA experiments do not consistently differ from those observed in DDA control method experiments. However TDA/DDA produces more consistent quantification of targeted proteins across technical replicate experiments relative to DDA control method experiments (i.e. more filled boxes with error bars in Figure S3), and therefore increases the potential to perform statistical analysis of fold-changesfor targeted proteins following [Ting, et al. (2009](#_ENREF_38)), as is discussed in detail in the main text.

**References**

Kalli A, Smith GT, Sweredoski MJ, Hess S. 2013. Evaluation and optimization of mass spectrometric settings during data-dependent acquisition mode: focus on LTQ-Orbitrap mass analyzers. Journal of Proteome Research.12:3071-3086.

Reis RS, Hart-Smith G, Eamens AL, Wilkins MR, Waterhouse PM. 2015a. Gene regulation by translational inhibition is determined by Dicer partnering proteins. Nature Plants.1.

Ting L, Cowley MJ, Hoon SL, Guilhaus M, Raftery MJ, Cavicchioli R. 2009. Normalization and statistical analysis of quantitative proteomics data generated by metabolic labeling. Molecular & Cellular Proteomics.8:2227-2242.
